# Generation of novel cationic antimicrobial peptides from natural non-antimicrobial sequences by acid-amide substitution

**DOI:** 10.1186/1476-0711-10-11

**Published:** 2011-03-22

**Authors:** Satoshi Ueno, Masaomi Minaba, Yuji Nishiuchi, Misako Taichi, Yasushi Tamada, Toshimasa Yamazaki, Yusuke Kato

**Affiliations:** 1 National Institute of Agrobiological Sciences, Oowashi 1-2, Tsukuba, Ibaraki 305-8634, Japan; 2 SAITO Research Center, Peptide Institute, Inc., Ibaraki, Osaka 567-0085, Japan

## Abstract

**Background:**

Cationic antimicrobial peptides (CAMPs) are well recognized to be promising as novel antimicrobial and antitumor agents. To obtain novel skeletons of CAMPs, we propose a simple strategy using acid-amide substitution (i.e. Glu→Gln, Asp→Asn) to confer net positive charge to natural non-antimicrobial sequences that have structures distinct from known CAMPs. The potential of this strategy was verified by a trial study.

**Methods:**

The pro-regions of nematode cecropin P1-P3 (P1P-P3P) were selected as parent sequences. P1P-P3P and their acid-amide-substituted mutants (NP1P-NP3P) were chemically synthesized. Bactericidal and membrane-disruptive activities of these peptides were evaluated. Conformational changes were estimated from far-ultraviolet circular dichroism (CD) spectra.

**Results:**

NP1P-NP3P acquired potent bactericidal activities via membrane-disruption although P1P-P3P were not antimicrobial. Far-ultraviolet CD spectra of NP1P-NP3P were similar to those of their parent peptides P1P-P3P, suggesting that NP1P-NP3P acquire microbicidal activity without remarkable conformational changes. NP1P-NP3P killed bacteria in almost parallel fashion with their membrane-disruptive activities, suggesting that the mode of action of those peptides was membrane-disruption. Interestingly, membrane-disruptive activity of NP1P-NP3P were highly diversified against acidic liposomes, indicating that the acid-amide-substituted nematode cecropin pro-region was expected to be a unique and promising skeleton for novel synthetic CAMPs with diversified membrane-discriminative properties.

**Conclusions:**

The acid-amide substitution successfully generated some novel CAMPs in our trial study. These novel CAMPs were derived from natural non-antimicrobial sequences, and their sequences were completely distinct from any categories of known CAMPs, suggesting that such mutated natural sequences could be a promising source of novel skeletons of CAMPs.

## Background

Cationic antimicrobial peptides (CAMPs) are well recognized to be promising as novel antimicrobial and antitumor agents. Natural CAMPs are structurally much diverse (e.g., linear cationic α-helical CAMPs, those enriched for specific amino acids, and those containing disulphide bonds and stable β-sheets) [1]. Because each category exhibits characteristic antimicrobial properties, numerous researchers have tried to identify novel skeletons of natural CAMPs from various organisms over the last two decades. Many categories of CAMPs were identified in the last century. However, most CAMPs which were recently found were categorized in known groups, suggesting that the search for novel skeletons may have almost reached its limit. To find novel skeletons, non-natural sequences have been also explored [2]. A major trial is combinatorial chemistry or similar approach such as construction of random peptide libraries and high-throughput screening [3-9]. Although these approaches produced some novel CAMPs, they are effective only for short sequences. However, several major categories of CAMPs are larger peptides that contain specific higher order structures as mentioned above. Introduction of non-natural peptide mimics is an alternative strategy [10-12]. This is an excellent strategy which can overcome the limitations inherent to peptides physical characteristics. A demerit of this strategy is that those peptide mimics cannot be prepared as recombinant products by ribosomal syntheses which can produce natural CAMPs.

In this study, we propose a simple strategy to obtain novel larger skeletons of CAMPs which only contain natural amino acids using acid-amide substitution (i.e. Glu→Gln, Asp→Asn), as described below.

(1) CAMPs are basic molecules. The positive charge is essential for antimicrobial activity, but most natural proteins/peptides are not basic.

(2) Rather than being limited to a specific skeleton, natural CAMPs are structurally diverse. Novel skeletons of CAMPs can be screened from modified nonbasic sequences that have structures distinct from known CAMPs by acquisition of net positive charge.

(3) As such nonbasic parent sequences, natural non-antimicrobial peptides or parts of proteins can be used.

(4) Substituting acidic residues with counterpart amides (i.e. Glu→Gln, Asp→Asn) can neutralize negative charges and confer net positive charge if the parent sequences contain substantial basic residues. Such substitution is "conservative," which minimally affects the unique structure of parent sequences in most cases. Introduction of basic residues at random positions is unfavorable because the sequences become uniform with increasing substitution and the unique structures of parent sequences are lost.

**Figure 1:**
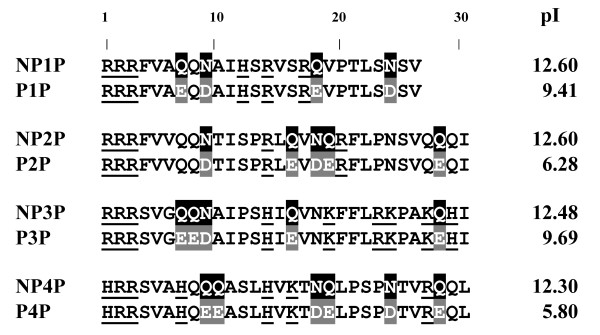
**Sequence alignment**. NP1P-NP4P are aligned with their parent sequences (P1P-P4P). Glu→Gln and Asp→Asn substitutions are indicated as inverted characters. Basic residues are underlined. The calculated pI is represented on the right side of each sequence.

As a trial study, the pro-regions of nematode cecropins were selected as parent sequences. Nematode cecropins are natural antimicrobial peptides [13-15]. Four nematode cecropins (P1-P4) have been identified in the pig roundworm, *Ascaris suum *[15]. A pro-region (26-30 residues long) is conserved at the C terminus of nematode cecropin precursors (Figure 1)[15]. It contains 4-6 acidic residues that might interact with mature peptides to suppress their antimicrobial activity in the precursor form [16]. Basic residues including a unique tribasic site, (H/R)RR, are also present at the N terminus. These sequences are expected to be promising parent sequences because the pro-regions have been estimated to acquire strong net positive charges by acid-amide substitution.

## Methods

### Peptides

Peptides used in this study were prepared at Biologica Co., (Nagoya, Japan). Briefly, the peptides were synthesized by the Fmoc method, and purified by reverse phase HPLC. The products were confirmed by time-of-flight mass spectrometry on a Voyager DE Mass Spectrometer, Applied Biosystems (Foster city, CA, USA).

### Microorganisms

*E. coli *JM109 was purchased from Takara (Otsu, Japan). *Saccharomyces cerevisiae *MAFF113011 was obtained from the National institute of Agrobiological Sciences, Ibaraki, Japan. Other strains described below were transferred from the National Institute of Technology and Evaluation, Kazusa, Japan: *Staphylococcus aureus *IFO12732, *Bacillus subtilis *IFO3134, *Micrococcus luteus *IFO12708, *Pseudomonas aeruginosa *IFO3899, *Salmonella typhimurium *IFO13245 and *Serratia marcescens *IFO3736.

### Microbicidal assay

Microbicidal assay was performed as previously described [17]. A low ionic strength condition was used to detect bactericidal activity with higher sensitivity. Briefly, each microbial strain in the mid-exponential phase was suspended in 10 mM Tris/HCl, pH 7.5. The microbial suspension was mixed with peptides. After 2 h incubation, the suspension was diluted 1,000 times and inoculated on to plates of IFO702 medium (1% polypeptone, 0.2% yeast extract, 0.1% MgSO_4 _7H_2_O, 2% agar). The numbers of colonies were counted, and a plot of peptide concentration vs colony number was created.

### Hemolytic assay

Hemolytic assay was performed as previously described [18]. Human erythrocytes were used. Hemolysis was estimated as the leakage of haemoglobin. The erythrocytes were washed and resuspended in 10 mM Tris-HCl, pH 7.5, containing 308 mM sucrose. The erythrocyte suspension was diluted by 30%. The diluted suspension was mixed with the equal volume of peptide solution dissolved in the same buffer. After 0.5 h of incubation, the test suspension was centrifuged to remove the intact erythrocytes. The supernatant was diluted, and A_540 _was measured. The hemolysis caused by pure water was defined as 100% lysis. Amphotericin B was used as a positive control.

### Cytoplasmic membrane permeability assay

Cytoplasmic membrane permeabilization of *S. aureus *was determined with a voltage-sensitive dye, diS-C_3_-(5) [19]. Bacteria in the mid-exponential phase were suspended in 10 mM Tris-HCl, pH 7.5 to an OD_600 _of 0.05. Changes in fluorescence were continuously monitored using a RF-5300PC spectrofluorometer (Shimadzu, Kyoto, Japan) at an excitation wavelength of 622 nm and an emission wavelength of 670 nm. The bacterial suspension was incubated with 400 nM diS-C_3_-(5). Test peptides were added to the bacterial suspension after the dye uptake was maximal. The maximal increase in fluorescence due to disruption of the cytoplasmic membrane was recorded.

### Liposome disruption assay

Membrane-disrupting activity was estimated by liposome disruption assay [19]. Bovine heart phosphatidylglycerol (PG), egg cardiolipin (CL), and egg phosphatidylcholine (PC) were purchased from Avanti Polar Lipids, Inc., Alabama, USA. A lipid film was prepared by rotary evaporation of lipid solution (1 mg lipid in 1 ml chloroform). The lipid film was hydrated with 1 ml of 10 mM Tris-HCl buffer (pH 7.5) containing 75 mM calcein. Lipid dispersions were sonicated and subjected to five freeze-thaw cycles. Non-trapped calcein was removed by gel filtration on a Sephacryl S-300 spin column (GE Healthcare Bio-Science Corp., Piscataway, NJ, USA) equilibrated with 10 mM Tris-HCl (pH 7.5) containing 175 mM NaCl and 1 mM EDTA. These calcein-entrapped liposomes were diluted at a ratio of 1:1000 in 10 mM Tris-HCl (pH 7.5) containing 350 mM sucrose. Calcein release after membrane disruption was evaluated by measuring fluorescence intensity at 515 nm with excitation at 492 nm on a RF-5300PC spectrofluorometer at room temperature.

### Circular dichroism (CD) spectroscopy

CD spectra were recorded on a J-720 spectropolarimeter (JASCO, Tokyo, Japan) in the far-ultraviolet (UV) range from 190 to 260 nm using a 0.1 cm thermostatted cell at 25°C. Peptides were dissolved in 10 m*M *Tris-HCl (pH 7.5) containing tri-fluoroethanol (TFE) at 0-100%. For measurement under the presence of liposomes, peptides were dissolved at 100 μg/ml containing 500 μg/ml liposomes at 20°C.

## Results and Discussion

### Microbicidal activities

The pro-region of cecropin P1-P4 (P1P-P4P) and the acid-amide-substituted mutants (acidic residues neutralized P1P-P4P: NP1P-NP4P) were chemically synthesized. The C-terminal-COOH was not modified. The minimum bactericidal concentrations (MBCs) for these peptides were estimated (Table 1). Parent peptides (P1P-P4P) were not bactericidal at ≤300 μg/ml except for weak activities of P3P. These results were expected from the inhibitory effects of pro-regions to the activity of mature peptides [16]. NP1P-NP3P displayed bactericidal activity against all tested Gram-positive bacteria (*S. aureus *IFO12732, *B. subtilis *IFO3134, and *M. luteus *IFO12708), some Gram-negative bacteria (*P. aeruginosa *IFO3899, *S. typhimurium *IFO13245, and *E. coli *JM109), and a yeast (*S. cerevisiae *MAFF113011). The Gram-negative bacterium, *S. marcescens *IFO3736, was not susceptible. NP3P was the most effective among these peptides (e.g., MBC = 5 μg/ml against *S. aureus *IFO12732). The MBCs of NP1P-NP3P were comparable with those of mature nematode cecropins, which are well-recognized, potent, natural CAMPs [15]. These results suggest that the acid-amide substitution effectively confers bactericidal activities to some tested parent peptides. Only NP4P was not bactericidal at ≤300 μg/ml against all the tested microbes although enhancer activity for membrane-disruptive antimicrobial peptides was detected [19].

**Table 1:** Minimum bactericidal concentrations (MBCs) of NP1P-NP4P and their parent peptides.

Microbes	MBC, μg/ml			
	NP1P (P1P)	NP2P (P2P)	NP3P (P3P)	NP4P
**Gram-positive bacteria**				
***Staphylococcus aureus *IFO12732**	10 (>300)	5 (>300)	5 (>300)	>300 (>300)
***Bacillus subtilis *IFO3134**	30 (>300)	70 (>300)	20 (>300)	>300 (>300)
***Micrococcus luteus *IFO12708**	30 (>300)	30 (>300)	10 (200)	>300 (>300)
**Gram-negative bacteria**				
***Pseudomonas aeruginosa *IFO3899**	20 (>300)	70 (>300)	20 (70)	>300 (>300)
***Salmonella typhimurium *IFO13245**	30 (>300)	200 (>300)	20 (>300)	>300 (>300)
***Serratia marcescens *IFO3736**	>300 (>300)	>300 (>300)	>300 (>300)	>300 (>300)
***Escherichia coli *JM109**	10 (>300)	30 (>300)	7 (>300)	>300 (>300)
**Yeasts**				
***Sacchoromyces cerevisiae *MAFF113011**	200 (>300)	100 (>300)	7 (>300)	>300 (>300)

### Conformational analyses

**Figure 2:**
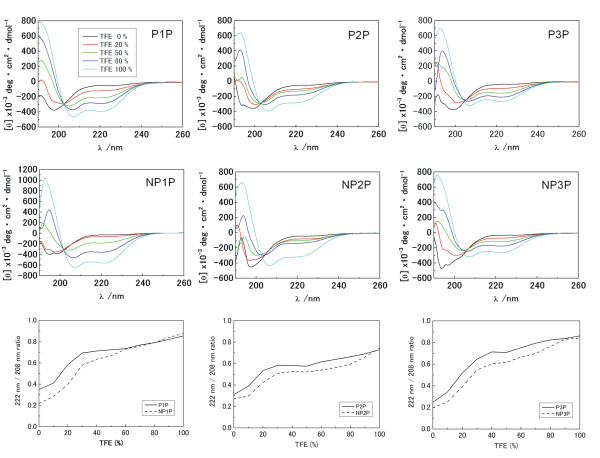
**TFE-dependent change of far-UV CD spectra**. NP1P-NP3P and P1P-P3P were examined. CD spectra in the presence of 0-100% TFE. The profiles of TFE-dependent change of A_222_/A_208 _are also represented.

Next, we examined if the acid-amide substitution would cause conformational changes by using CD spectroscopy (Figure 2). Far-UV CD spectra of NP1P-NP3P were similar to those of their parent peptides P1P-P3P at various concentration of TFE. Interestingly, the profile of TFE-dependent change of A_222_/A_208 _displayed a characteristic meta-stable phase around 50% TFE both for NP1P-NP3P and P1P-P3P series. These results suggest that NP1P-NP3P acquire microbicidal activity by acid-amide substitution without remarkable conformational changes. Since the acid-amide substitution is expected to be "structurally conservative", our strategy could be applicable for design of novel CAMPs from natural non-antimicrobial sequences which contain structures distinct from known CAMPs.

### Mode of action

**Figure 3:**
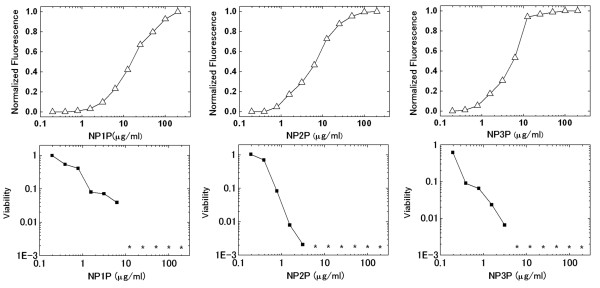
**Dose-membrane disruption and -bactericidal effect curve of NP1P-NP3P against *S. aureus *IFO12732**. These curves were simultaneously determined. The asterisks indicate that viable cells were not detected. Disruption of the cytoplasmic membrane was estimated by the increase in fluorescence intensity of diS-C**_3_**-(5). Changes in fluorescence were normalized by the value at the plateau of the dose-response curves.

To elucidate the mode of action against living microbial cells, the relationship between membrane-disruption and bacterial killing activities of NP1P-NP3P was assessed for *Staphylococcus aureus *IFO12732 (Figure 3). NP1P-NP3P destroyed *S. aureus *membrane at <1/10 concentration of MBC although P1P-P3P were neither membrane-disruptive nor bactericidal. Moreover, these peptides killed bacteria in almost parallel fashion with their membrane disruption activities. These results suggest that the bactericidal activities of all the NP1P-NP3P peptides against *S. aureus *are accomplished via membrane-disruption. In contrast, only slight haemolytic activities were detected for NP1P-NP3P, suggesting that the membrane disruptive activities were selective against microbial membranes (Table 2).

**Table 2:** Hemolytic activity of NP1P-NP3P and their parent peptides.

Agent	Concentration (μg/mL)	Hemolytic activity (%)
**NP1P (P1P)**	300	1.32 ± 0.02 (0.00 ± 0.00)
**NP2P (P2P)**	300	3.48 ± 0.01 (0.00 ± 0.01)
**NP3P (P3P)**	300	2.61 ± 0.02 (1.69 ± 0.05)
**amphotericin B**	30	82.5 ± 7.2

### Membrane-disruptive activities against acidic-liposomes

**Figure 4:**
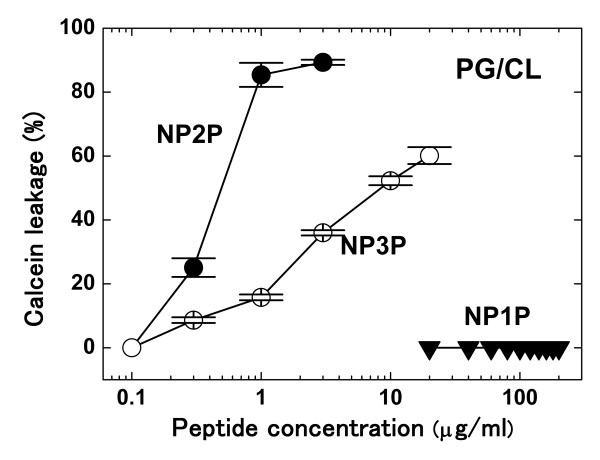
**Membrane-disruption against acidic-liposomes**. Calcein release from vesicles consisting of PG:CL = 3:1 was measured. Each point was obtained from three independent trials using a single preparation of liposomes and peptides, and represents the mean ± standard error of the mean.

Furthermore, membrane-disruptive activities against liposomes were also estimated. A liposome dye-leakage assay was used. P1P-P3P did not cause the dye-leakage at ≤300 μg/ml against the liposome membrane [molar ratio of phosphatidylglycerol (PG):cardiolipin (CL) was 3:1], which was negatively charged and mimicked the Gram-positive cytoplasmic membrane, and the liposome membrane consisting of only phosphatidylcholine (PC), which was neutrally charged and mimicked eukaryotic membranes. Far-UV CD spectra of P3P were almost identical under the presence and absence of PG/CL liposomes, suggesting that those peptides were not inserted into hydrophobic regions of liposome membranes [20]. In contrast, NP2P and NP3P damaged the PG/CL liposome membrane at ≥0.3 μg/ml (Figure 4). The concentration was equal or lower than MBCs against Gram-positive bacteria, which were measured under identical ionic conditions. NP3P (100 μg/ml) was precipitated under the presence of higher concentration of PG/CL liposome (500 μg/ml) although P3P was not affected under the same condition, suggesting that NP3P acquired strong interaction with PG/CL membrane by acid-amide substitutions. Since CD spectra could not be measured, the conformation of NP3P in the presence of PG/CL membrane remains unclear. NP2P and NP3P did not affect PC liposome membrane (data not shown), suggesting that NP2P and NP3P selectively attack the negatively charged membrane as usually found in bacteria. This observation agreed with that those peptides did not destroy erythrocyte membranes. It is noteworthy that NP3P exhibited stronger bactericidal properties but weaker membrane disruptive activities against PG/CL membranes than those of NP2P, i.e., activities against PG/CL were not parallel to those against *S. aureus *membrane. In addition, NP1P did not exhibit membrane-disruptive activity in PG/CL and PC membranes at ≤200 μg/ml. These results suggest that membrane-disruptive activities of NP1P-NP3P are highly diversified against PG/CL membranes although all of those peptides are strongly harmful against *S. aureus *membrane. The nematode cecropin pro-region is expected to be a unique and promising skeleton for novel synthetic CAMPs with diversified membrane-discriminative properties, i.e., CAMPs which exhibit distinct target-selectivity may be generated using this skeleton.

## Conclusions

The acid-amide substitution successfully generated some novel CAMPs in our trial study. These novel CAMPs were derived from natural non-antimicrobial sequences, and their sequences were completely distinct from any categories of known CAMPs. In addition, remarkable conformational changes were not detected between parent and substituted peptides although further study was necessary to estimate the conformation in the presence of membranes. This observation suggested that acid-amide substitution was a promising method to generate novel skeletons of CAMPs from natural non-antimicrobial sequences that have structures distinct from known CAMPs. This platform should not be limited to acid-amide substitution or natural parent sequences. Combination with limited introduction of basic residues and/or use of chimera sequences with some natural sequences might expand the flexibility of this platform.

Acid-amide substitution occurs at a high frequency in the mutations of natural proteins [21]. Such substituted residues could be accommodated in the structure and function of the parent proteins. The accumulation of acid-amide substitutions and gain of net positive charge could occasionally confer antimicrobial activities. This is a presumable scenario for the birth of natural CAMPs.

## Competing interests

The authors declare that they have no competing interests.

## Authors' contributions

SU and MM carried out the microbicidal assays, hemolytic assays, cytoplasmic membrane permeability assays, and liposome disruption assays. YT participated in the liposome disruption assays. TY, YN, and MT carried out the CD spectroscopy experiments. YK conceived of the study, participated in its design and coordination, and drafted the manuscript. All authors read and approved the final manuscript.
